# Cesarean Section

**Published:** 1910-11

**Authors:** 


BUFFALO MEDICAL JOURNAL

A Monthly Review of Medicine and Surgery

*EDITOR*

WILLIAM WARREN POTTER, M. D.

All communications, whether of a literary or business nature, books for review and exchanges, should be addressed to the editor, 238 Delaware Avenue, Buffalo, N.Y.

Vol. Lxvi. NOVEMBER, 1910. No. 4

### Cesarean Section

NO major obstetrical procedure claims the attention of obstetricians and of obstetrical surgeons at the present time, to the degree, perhaps, that Cesarean Section does. Though it is a method of delivery accorded the parturient woman in the earliest times, and has been handed down almost to the present time as a procedure to be invoked under extreme conditions, or when the natural power and channel become inadequate to accomplish delivery, it yet has not been made to take its true place among the surgical ways and means of the lying-in room to be readily available whenever it becomes hopefully needful. At all events, not until just now has it been made to assume a place alongside the other abdominal operations, competing with them for the same low percentages of mortality.

In the period of time before anesthetics and before Lister had wrought his great revolution, the operation offered little chance for the woman and small credit to the doctor. Now, however, all this is changed. Under aseptic conditions, with hospital advantages, an experienced surgeon, and intervention before damage has been done by prolonged labor, rupture of membranes for a long period, and repeated attempts at vaginal delivery, Cesarean section may be done in the confident hope of saving both mother and child.

Dr. Asa B. Davis, of the New York Lying-in Hospital, in a paper read at the recent meeting of the American Association of Obstetricians and Gynecologists held at Syracuse, N. Y., set forth the history and present status of Cesarean section most interestingly as well as instructively. It is a paper that deserves the study not only of every obstetric surgeon, but of every physician who practises obstetrics, much or little. Dr. Davis has had a large experience, having himself made the operation 78 times,—a greater number probably than any other operator. Such preparation entitles him to speak from the chair on the subject of Cesarean section.

He gives some interesting statistics in his paper[1]—among which we refer to a few. At the lying-in hospital, in the service of 68.200 obstetric patients, Cesarean Section was done 256 times; and of this number, 220 mothers and 207 children survived,—a maternal mortality of 14.06 per cent., and a fetal mortality of 20.08 per cent. Of the seventy-eight operations done by Dr. Davis, three were performed in tenements, the uterus being delivered; since August 12, 1903, he has done the section seventy-five times, in none of which has the uterus been delivered. He operated on sixty-seven women up to the date of his paper, twelve of whom had repeated sections, ten twice, one three times, and one five times, the third and fourth done by himself; of the one three times, the second operation was done by the author. During the nine months preceding the reading of his paper the author had performed Cesarean Section eighteen times, fifteen mothers and thirteen children surviving.

Dr. Davis does not feel it just to render these women sterile, hence does not hesitate to perform Cesarean section repeatedly on the same woman, particularly since the operation, after the first, usually can be made one of election as to time and place. He advocates the small high median incision, that is to say, one entirely above the umbilicus.

What Dr. Davis says in relation to the operation itself is of sufficient interest to justify reference to his statement in considerable detail. While in a large proportion of cases, the operator has no choice, the women already being in labor, or in conditions of emergency, yet in those patients under observation before labor, he advises to wait until labor actually sets in, accepting Nature's law as to maturity of child and other favoring conditions; then to operate without delay. The patient is prepared as for any other abdominal section being fully anesthetised, on the table in the horizontal posture. The abdomen is opened by an incision 8 to 10 cm. long from above down to the umbilicus, and gauze pads wet in warm salt solution immediately are placed above the fundus to hold back the omentum and intestines. An assistant makes pressure against the side walls of the abdomen, continuing it until the uterus is emptied and partly closed by sutures. By a few strokes of the knife, the uterus is opened, the hand is passed in and swept between the membranes and uterine walls; or, if the placenta presents it is either pushed aside or perforated.

1. To be published, as the other papers read at that meeting will be, in an early number of the *American Journal of Obstetrics;* also in the Transactions of the Association.

As the hand is withdrawn it grasps the anterior thigh or breech, doing extraction and in case of a presenting breech podalic version. The cord is clamped in two places and cut by an assistant, who takes the child away and cares for it. A bullet forceps grasps the uterine wall at the upper and lower angles of the wound, the placenta and membranes are extracted, and the uterus cleared of clots. If hemorrhage is profuse, a sterile towel is quickly packed into the cavity. Now the uterus is closed by six or eight deep interrupted sutures of No. 2 chromic gut, these being buried by a continuous suture of like material,—a deep Lembert stitch,—the packing being gradually withdrawn during this process. The abdomen is closed in three layers, a dressing is held in place by tight adhesive straps over the wound, across which an abdominal binder is pinned tight,—loose elsewhere. The head of the bed is raised, to favor drainage and the descent of the uterus. The after-treatment differs in no way from that of an ordinary abdominal operation or postpartum woman, unless there should be complications to deal with.

Dr. Davis says though he regards the operation as serious, even dangerous, it is no more so than any other abdominal section, and it should not be singled out as one to be performed in great haste. It should be done with the same deliberation required in all operations within the abdomen, there being danger in doing otherwise. Several times he has found intestine in front of the uterus; once it was injured. Commenting on the small high incision he says it should be long enough to permit easy delivery of the fetus, but the abdominal wall at this point at term is thin, hence stretches easily. This incision does not allow easy exposure or escape of the abdominal contents, not infrequently all that is seen being the uterus and a small portion of omentum. Moreover, this wound is away from the area of greatest strain upon the abdominal wall, and at a point reinforced by the recti muscles as they approach each other toward their upper attachments. He has never seen hernia following it and, he affirms, that the liability to adhesion between the abdominal and uterine wounds is greatly diminished.

Dr. Davis in this paper has made the most important contribution to the study of Cesarean section that has appeared in the recent literature of the subject. It is the offering of a master, not of the theorist; his statements though modest are based upon a larger experience with the operation than any other man, thus justly entitling him to be regarded as a specialist in this method of delivering the parturient woman. His paper will modify opinions and methods relating to the classic Cesarean section.

